# Optimal Filters with Multiple Packet Losses and its Application in Wireless Sensor Networks

**DOI:** 10.3390/s100403330

**Published:** 2010-04-06

**Authors:** Yonggui Liu, Bugong Xu, Linfang Feng, Shanbin Li

**Affiliations:** College of Automation Science and Engineering, South China University of Technology, Guangzhou, 510640, China; E-Mails: aubgxu@scut.edu.cn (B.X.); fenglinfang2009@gmail.com (L.F.); lishb@scut.edu.cn (S.L.)

**Keywords:** packet losses, optimal estimation, wireless sensor networks, Kalman filter, minimum variance filter

## Abstract

This paper is concerned with the filtering problem for both discrete-time stochastic linear (DTSL) systems and discrete-time stochastic nonlinear (DTSN) systems. In DTSL systems, an linear optimal filter with multiple packet losses is designed based on the orthogonal principle analysis approach over unreliable wireless sensor networks (WSNs), and the experience result verifies feasibility and effectiveness of the proposed linear filter; in DTSN systems, an extended minimum variance filter with multiple packet losses is derived, and the filter is extended to the nonlinear case by the first order Taylor series approximation, which is successfully applied to unreliable WSNs. An application example is given and the corresponding simulation results show that, compared with extended Kalman filter (EKF), the proposed extended minimum variance filter is feasible and effective in WSNs.

## 1. Introduction

In wireless networks, data losses, communication delay and constrained bandwidth are general problems across communication links because of collision and transmission errors. Especially, in WSNs, due to its limited resources, such as restricted computation, processing and communication ability, data/packet losses should be further studied.

Many researchers are interested in networked control systems with packet losses. The research on packet losses can be traced back to Nahi [1] and Hadidi [2]. Recently, the packet loss problem has been studied using jump linear systems (JLS), which is a hybrid system with model transitions modeled as Markov chains that switches among several discrete models [3–5]. JLS methods restrict their formulation to the steady-state case, where Kalman gain is constant. Furthermore, the transition probability and state error covariance matrices are required being computed exactly [6]. Sinopoli *et al.* [7] consider general case of time varying Kalman gain and discusses how packet dropouts can affect state estimation. They illustrate that there exists a certain threshold of the packet dropout rate. Under packet dropouts, their filter has a smaller linear minimum mean square error than static counterpart. Liu *et al.* [8] extend the idea to the case where there are multiple sensors and packets from different sensors dropping independently. In practice, it is assumed that packets are dropped independently, which is certainly not true in case where burst packets are dropped or in queuing networks where consecutive packets are not dropped [7,8]. They also use the expected value of the error covariance matrix as the measure of performance. This might ignore the fact that events with arbitrarily low probability can make the expected value diverge. So Epstein *et al.* [9] give a more complete characterization of the estimator performance instead of considering a probabilistic description of the error covariance. The optimal filtering problem is considered for systems where multiple packets are dropped in an unreliable network [10–12]. Different from [10–12], where only the multiple packet dropouts are considered, reference [13] investigates both the estimation problem for systems with bounded random measurement delays and packet dropouts, which are described by some binary random variables whose probabilities are only known. Schenato [14] proposes a probabilistic framework to design the minimum error covariance estimator in a generic digital communication network where sensor's observation packets are subject to random delays and packet losses. Speranzon *et al.* [15] analyze and design a distributed adaptive algorithm to estimate a time-varying signal, measured by WSNs, where measurement noise and packet losses are considered. But they do not consider multiple packet losses in WSNs.

On the other hand, mobile target tracking with multiple sensors measurement is an important application of WSNs in recent years. There are great deals of wireless sensor nodes deployed randomly in a monitored field. One node or several nodes are scheduled as tasking nodes in target tracking application at each time step. Some natural problems are that how to apply filters to WSNs and who are scheduled as current task sensor nodes. There are many sensor scheduling strategies, such as the nearest distance scheduling, where the nearest sensor node to the target is scheduled as task node, minimum trace scheduling [17], where minimum trace sensor node of the error covariance matrix is scheduled, adaptive sensor scheduling [18], which selects the next tasking sensor and determines sampling interval according to the predicted accuracy and tracking cost. We propose an improved dynamic-grouping scheduling strategy (DGSS) which considers not only energy consumption and predicted accuracy, but also the real-time property of tracking target.

In this paper, we discuss minimum variance filters (MVFs) with multiple packet losses for systems that are considered not only DTSL systems but also DTSN systems in WSNs. The MVFs with packet losses across an unreliable network are designed and packet losses are assumed to be random with a given i.i.d distribution. Unlike [14] and [16], where the estimator is computed depending on whether the current measurement is received, our MVFs can be computed only depending on the packet arrival rate *p_k_* at each time instant and do not need know if a measurement is received at a particular time instant. Furthermore, our filters do not require that the measurement is time-stamped.

Simulation results show that it is feasible and effective that DGSS is adopted to select next sensor node as task node, and MVFs with multiple packet losses are used to track mobile target.

The remainder of the paper is organized as follows. MVFs with multiple packet losses are formulated in Section 2. The linear MVF is designed and a numerical example shows that linear MVF is effective in Section 3. The nonlinear MVF is derived and a target tracking example is shown in WSNs in Section 4. Finally, some conclusions are drawn in Section 5.

## 2. Problem Formulation

In WSNs, mobile target tracking with multiple sensors measurement is an important application in recent years. In practice, sensor measurements are probably lost. How to deal with packet losses and how to make multiple sensors collaborate to complete common task? We are interested in these problems and discuss them in the following part.

**Figure 1.**
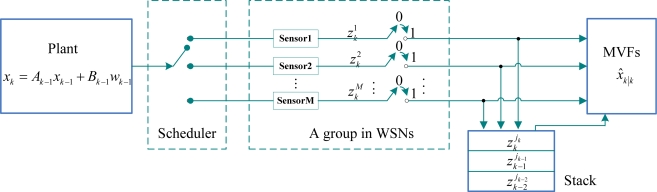
MVFs with Multiple Packet Losses and scheduling in WSNs.

In Figure 1, we assume that measurements from the plant are encapsulated into packets, but are not time-stamped, and then transmitted through WSNs, whose goal is to deliver packets from a plant to a filter.

In the same time instant, the scheduler selects only one sensor from *N* sensors to sample measurements according to sensor scheduling strategies, where measurements come probably from the same sensor, also come probably from different sensor at different time step. At the filter side, if the current measurement has one or more delays, or is lost, the filter utilizes the latest measurement in the stack to update the state estimation, where the stack retains the latest three measurements. The stack has property as follows: last input first output. For example, the order of measurements entering stack is $z_{k-2}^{j_{k-2}}$, $z_{k-1}^{j_{k-1}}$, $z_{k}^{j_{k}}$ from a group in WSNs. While the order of coming out is $z_{k}^{j_{k}}$, $z_{k-1}^{j_{k-1}}$, $z_{k-2}^{j_{k-2}}$, respectively. Therefore, MVFs can use the latest measurement from the stack in Figure 1. If the current measurement is correctly received in time, the filter uses directly measurement received from a group in WSNs (About a group, please refer to Subsection 4.2.1 in p.12). In addition, we assume that packet losses are uncorrelated and there is not a retransmission in order to increase performance of real time once a packet is lost.

According to the above framework in Figure 1, we consider a time-varying DTSL system as follows:

(1a) $$x_{k+1}=A_{k}x_{k}+B_{k}w_{k}$$

(1b) $$z_{k}^{j_{k}}=C_{k}^{j_{k}}x_{k}+v_{k}^{j_{k}}$$

(1c) $$y_{k}=\gamma_{k}z_{k}^{j_{k}}+(1-\gamma_{k})\gamma_{k-1}z_{k-1}^{j_{k-1}}+(1-\gamma_{k})(1-\gamma_{k-1})z_{k-2}^{j_{k-2}}$$

where *x_k_* ∈ *R^n^* is the state vector at time step *k*; *j_k_* is sensor node number at time step *k*; $z_{k}^{j_{k}}$, $z_{k-1}^{j_{k-1}}$, and $z_{k-2}^{j_{k-2}}$ are measurement outputs of sensor node *j_k_*, *j*_*k*−1_, and *j*_*k*–2_ at time step *k*, *k*−1, and *k*−2 respectively; *y_k_* is measurement received by the filter at time step *k*; *k*=1,2,…,*M*, where *M* is total sensor number in a group; *A_k_*, *B_k_* and $C_{k}^{j_{k}}$ are time-varying linear matrices with appropriate dimensions respectively; *w_k_* is state noises with variance *Q_k_* and $v_{k}^{j_{k}}$ is measurement noises of sensor node *j_k_* with variance $R_{k}^{j_{k}}$ at time step *k*; *γ_k_* is a random variable taking value 0 or 1, where 0 stands for packet loss and 1 stands for received packet. The probability is as follows, respectively:

(2) $$\mathrm{Pr}[\gamma_{k}=1]=p_{k}$$

(3) $$\mathrm{Pr}[\gamma_{k}=0]=1-p_{k}$$

Taking expectation:

(4) $$E\gamma_{k}=p_{k}$$

(5) $$E\gamma_{k}\gamma_{i}=p_{k}p_{i},k\neq i$$

(6) $$E\gamma_{k}^{2}=p_{k}$$

From (1c) we know that $z_{k}^{j_{k}}$ is lost if *γ_k_* = 0 at time step *k*, and *y_k_* depends on *γ*_*k*−1_, where $y_{k}=z_{k-1}^{j_{k-1}}$, if *γ*_*k*−1_ =1. Otherwise $y_{k}=z_{k-2}^{j_{k-2}}$.

Given the state vector *x*_*k*+1_ defined by (1a). It is desired to find the estimate of *x*_*k*+1_, denoted by *x̂*_*k*+1|*k*+1_, which is a linear function of observations *y*_0_,…,*y*_*k*+1_ minimizing:

(7) $$E_{x}E_{\gamma }{\left[x_{k+1}-{\hat{x}}_{k+1|k+1}\right]}^{T}L[x_{k+1}-{\hat{x}}_{k+1|k+1}]$$

where *L* is a symmetric positive definite matrix; *y_k_* is given by (1c), *E_x_* is the expectation with respect to *x_k_*, *w_k_*, and *v_k_*; and *E_γ_* is the expectation with respect to *γ*.

Since state estimation *x̂*_*k*+1|*k*+1_ is a linear function of *y*_0_, *y*_1_,…, and *y*_*k*+1_, it can be written as:

(8) $${\hat{x}}_{k+1|k+1}=\sum_{i=0}^{k+1}a_{i}y_{i}$$

where *a_i_* is an *n* × *m* matrix.

Our objective is to design MVFs *x̂*_*k*+1|*k*+1_ based on received measurement sequences *y*_0_, *y*_1_,…, *y*_*k*+1_. According to (1c), we know *γ*_0_, *γ*_1_,..., *γ*_*k*+1_ are unknown stochastic variable sequences. That is, we desire to find *E*[*x*_*k*+1_ | *y*_*k*+1_, *y_k_*, *y*_*k*−1_,..., *y*_0_].

**Definition:** Error covariance matrix and state variance matrix at the time *k* respectively are defined as follows:

Error covariance matrix:

(9a) $$P_{k|k}\equiv E_{x}E_{\gamma }{\left[x_{k}-{\hat{x}}_{k|k}\right]}^{T}[x_{k}-{\hat{x}}_{k|k}]$$

and state variance matrix:

(9b) $$S_{k}\equiv E[x_{k}^{T}x_{k}]$$

**Assumption 1**: For systems (1a) and (1b), *w_k_* and *v_k_* are uncorrelated white noises with zero means and variances *Q_k_* and *R_k_* respectively. Furthermore, if *k* ≠ *j*, *E*[*w_k_w_j_*] = 0, otherwise *E*[*w_k_w_j_*] = *Q_k_*, and *Q_k_* > 0; if *k* ≠ *j E*[*v_k_v_j_*] = 0, otherwise E[*v_k_v_j_*] = *R_k_*, and *R_k_* > 0.

**Assumption 2**: The initial state *x*_0_ is independent of *w_k_* and *v_k_* with:

$$Ex_{0}=m_{0},\;E[x_{0}^{T}x_{0}]=S_{0},\;E{\left[x_{0}-m_{0}\right]}^{T}[x_{0}-m_{0}]=P_{0}.$$

## 3. Linear Minimum Variance Filters with Multiple Packet Losses

In this section we will show our main results on linear minimum variance filters with multiple packet losses based on the orthogonal principle analysis approach. In time-varying DTSL systems, a linear filter with multiple packet losses is to be designed. Before giving our main results, firstly we will present the following Lemma.

**Lemma 1:**

(10) $$E_{x}E_{\gamma }[x_{k+1}-{\hat{x}}_{k+1|k+1}]y_{i}^{T}=0,\;\;\;i=0,1,\ldots ,k+1.$$

(11) $$P_{k+1|k+1}=E_{x}E_{\gamma }[x_{k+1}-{\hat{x}}_{k+1|k+1}]x_{k+1}^{T}$$

(12) $$E_{x}E_{\gamma }[{\hat{x}}_{k|k}y_{i}^{T}]=E_{x}E_{\gamma }[x_{k}y_{i}^{T}],\;i=0,1,\cdots ,k+1$$

**Proof**: Define estimation error: *x̃*_*k*+1_ ≡ *x*_*k*+1_ − *x̂*_*k*+1|*k*+1_. Because *x̃*_*k*+1_ and *y_i_* are orthogonal, *i* = 0,1,...,*k*+1, we attain easily (10) according to orthogonal principle [12].

Since *x̂*_*k*+1|*k*+1_ is a linear function of observations *y_i_*, *i*=0, 1,…, *k*, *k*+1. From (8) and (10) we may know:

$$E_{x}E_{\gamma }[x_{k+1}-{\hat{x}}_{k+1|k+1}]{\hat{x}}_{k+1|k+1}^{T}=0$$

From (9) it follows that:

$$\begin{array}{l} P_{k+1|k+1}=E_{x}E_{\gamma }[x_{k+1}-{\hat{x}}_{k+1|k+1}]{\left[x_{k+1}-{\hat{x}}_{k+1|k+1}\right]}^{T}\\ =E_{x}E_{\gamma }[(x_{k+1}-{\hat{x}}_{k+1|k+1})x_{k+1}^{T}-(x_{k+1}-{\hat{x}}_{k+1|k+1}){\hat{x}}_{k+1|k+1}^{T}]\\ =E_{x}E_{\gamma }[x_{k+1}-{\hat{x}}_{k+1|k+1}]x_{k+1}^{T} \end{array}$$

From (10) it follows that:

$$E_{x}E_{\gamma }[x_{k+1}y_{i}^{T}]=E_{x}E_{\gamma }[{\hat{x}}_{k+1|k+1}]y_{i}^{T},i=0,1,\cdots ,k+1$$

**Remark 1**: Substituting (8) and (10) into (7), we may know that (7) attains minimum value. That is, (10) is the sufficient condition for (7) taking minimum value.

### 3.1. Design of Linear Filter with Multiple Packet Losses

Utilizing the above background knowledge, we will design a linear minimum variance filter with multiple packet losses.

**Theorem 1**: For systems (1) satisfying Assumption 1 and 2, the linear minimum variance filter (LMVF) is shown as follows:

(13) $${\hat{x}}_{k+1|k+1}=F_{k+1}{\hat{x}}_{k|k}+K_{k+1}y_{k+1}$$

where:

(14) $$F_{k+1}=A_{k}-K_{k+1}J$$

(15) $$\begin{array}{l} K_{k+1}=[A_{k}P_{k|k}J^{T}+p_{k+1}B_{k}Q_{k}B_{k}^{T}{\left(C_{k+1}^{j_{k+1}}\right)}^{T}]\times [{\mathrm{JP}}_{k|k}J^{T}-{\mathrm{JS}}_{k}J^{T}+p_{k+1}C_{k+1}^{j_{k+1}}A_{k}S_{k}A_{k}^{T}{\left(C_{k+1}^{j_{k+1}}\right)}^{T}\\ +p_{k}(1-p_{k+1})C_{k}^{j_{k}}S_{k}{\left(C_{k}^{j_{k}}\right)}^{T}+(1-p_{k})(1-p_{k+1})C_{k-1}^{j_{k-1}}A_{k-1}^{-1}S_{k}A_{k-1}^{-T}{\left(C_{k-1}^{j_{k-1}}\right)}^{T}\\ +p_{k+1}R_{k+1}^{j_{k+1}}+p_{k}(1-p_{k+1})R_{k}^{j_{k}}+(1-p_{k})(1-p_{k+1})R_{k-1}^{j_{k-1}}\\ {+p_{k+1}C_{k+1}^{j_{k+1}}B_{k}Q_{k}B_{k}^{T}{\left(C_{k+1}^{j_{k+1}}\right)}^{T}+(1-p_{k+1})(1-p_{k})C_{k-1}^{j_{k-1}}A_{k-1}^{-1}B_{k-1}Q_{k-1}B_{k-1}^{T}{\left(A_{k-1}^{-1}\right)}^{T}{\left(C_{k-1}^{j_{k-1}}\right)}^{T}]}^{-1} \end{array}$$

(16) $$P_{k+1|k+1}=F_{k+1}P_{k|k}A_{k}^{T}+(I_{n}-p_{k+1}K_{k+1}C_{k+1}^{j_{k+1}})B_{k}Q_{k}B_{k}^{T}$$

(17) $$J=p_{k+1}C_{k+1}^{j_{k+1}}A_{k}+p_{k}(1-p_{k+1})C_{k}^{j_{k}}+(1-p_{k})(1-p_{k+1})C_{k-1}^{j_{k-1}}A_{k-1}^{-1}$$

(18) $$S_{k+1}=A_{k}S_{k}A_{k}^{T}+B_{k}Q_{k}B_{k}^{T}$$

Initial value *x̂*_0|0_ = *m*_0_, *P*_0|0_ = *P*_0_ and *S*_0_ = *P*_0_.

**Proof**: From the equation (1a) it follows that:

(19) $$x_{k-1}=A_{k-1}^{-1}x_{k}-A_{k-1}^{-1}B_{k-1}w_{k-1}$$

where it assumes *A*_*k*−1_ is invertible.

From (1) we can get:

(20) $$\begin{array}{l} y_{k+1}=\gamma_{k+1}z_{k+1}+(1-\gamma_{k+1})\gamma_{k}z_{k}+(1-\gamma_{k-1})(1-\gamma_{k})z_{k-1}\\ =\gamma_{k+1}[C_{k+1}^{j_{k+1}}A_{k}x_{k}+C_{k+1}^{j_{k+1}}B_{k}w_{k}+v_{k+1}^{j_{k+1}}]+(1-\gamma_{k+1})\gamma_{k}[C_{k}^{j_{k}}x_{k}+v_{k}^{j_{k}}]\\ +(1-\gamma_{k+1})(1-\gamma_{k})[C_{k-1}^{j_{k-1}}A_{k-1}^{-1}x_{k}-C_{k-1}^{j_{k-1}}A_{k-1}^{-1}B_{k-1}w_{k-1}+v_{k-1}^{j_{k-1}}] \end{array}$$

Let us substitute (1a), (13) and (20) into (10) respectively. Making use of (19), it follows that:

(21) $$\begin{array}{l} E_{x}E_{\gamma }[x_{k+1}-{\hat{x}}_{k+1|k+1}]y_{i}^{T}\\ =E_{x}E_{\gamma }\{A_{k}x_{k}+B_{k}w_{k}-F_{k+1}{\hat{x}}_{k|k}-K_{k+1}[\gamma_{k+1}C_{k+1}^{j_{k+1}}A_{k}x_{k}+(1-\gamma_{k+1})\gamma_{k}C_{k}^{j_{k}}x_{k}\\ +(1-\gamma_{k+1})(1-\gamma_{k})C_{k-1}^{j_{k-1}}A_{k-1}^{-1}x_{k}+\gamma_{k+1}v_{k+1}^{j_{k+1}}+(1-\gamma_{k+1})\gamma_{k}v_{k}^{j_{k}}+(1-\gamma_{k+1})(1-\gamma_{k})v_{k-1}^{j_{k-1}}\\ +\gamma_{k+1}C_{k+1}^{j_{k+1}}B_{k}w_{k}-(1-\gamma_{k+1})(1-\gamma_{k})C_{k-1}^{j_{k-1}}A_{k-1}^{-1}B_{k-1}w_{k-1}]\}\\ \times [\gamma_{i}C_{i}^{j_{i}}x_{i}+(1-\gamma_{i})\gamma_{i-1}C_{i-1}^{j_{i-1}}x_{i-1}+(1-\gamma_{i})(1-\gamma_{i-1})C_{i-2}^{j_{i-2}}x_{i-2}+\gamma_{i}v_{i}^{j_{i}}\\ {+(1-\gamma_{i})\gamma_{i-1}v_{i-1}^{j_{i-1}}+(1-\gamma_{i})(1-\gamma_{i-1})v_{k-2}^{j_{i-2}}]}^{T}=0,\;\;\;i=2,3,\ldots ,k+1;j_{i}=1,2,\ldots ,N \end{array}$$

Taking *i* = 2,3,…*k*−2, and making use of Assumption 1 and (12) in Lemma 1, the above equation follows that:

$$\begin{array}{l} E_{\gamma }\{A_{k}-F_{k+1}-K_{k+1}[\gamma_{k+1}C_{k+1}^{j_{k+1}}A_{k}+(1-\gamma_{k+1})\gamma_{k}C_{k}^{j_{k}}+(1-\gamma_{k+1})(1-\gamma_{k})C_{k-1}^{j_{k-1}}A_{k-1}^{-1}]\}\\ \;\;\;\;\;\times E_{x}\{x_{k}{\left[\gamma_{k-2}C_{k-2}^{j_{k-2}}x_{k-2}+(1-\gamma_{k-2})\gamma_{k-3}C_{k-3}^{j_{k-3}}x_{k-3}+(1-\gamma_{k-2})(1-\gamma_{k-3})C_{k-4}^{j_{k-4}}x_{k-4}\right]}^{T}\}=0 \end{array}$$

Because:

$$E_{x}\{x_{k}{\left[\gamma_{k-1}C_{k-1}^{j_{k-1}}x_{k-1}+(1-\gamma_{k-1})\gamma_{k-2}C_{k-2}^{j_{k-2}}x_{k-2}+(1-\gamma_{k-1})(1-\gamma_{k-2})C_{k-3}^{j_{k-3}}x_{k-3}\right]}^{T}\}\neq 0$$

we can obtain:

$$E_{\gamma }\{A_{k}-F_{k+1}-K_{k+1}[\gamma_{k+1}C_{k+1}^{j_{k+1}}A_{k}+(1-\gamma_{k+1})\gamma_{k}C_{k}^{j_{k}}+(1-\gamma_{k+1})(1-\gamma_{k})C_{k-1}^{j_{k-1}}A_{k-1}^{-1}]\}=0$$

and know easily that:

(22) $$\begin{array}{cc} F_{k+1} & =A_{k}-K_{k+1}[p_{k+1}C_{k+1}^{j_{k+1}}A_{k}+(1-p_{k+1})p_{k}C_{k}^{j_{k}}+(1-p_{k+1})(1-p_{k})C_{k-1}^{j_{k-1}}A_{k-1}^{-1}]\\ & =A_{k}-K_{k+1}J \end{array}$$

where (4)–(6) is used. Obviously, (14) and (17) can be attained by (22).

From Lemma 1 we take *i*=*k*+1 and then it follows that:

$$\begin{array}{l} E_{x}E_{\gamma }[x_{k+1}-{\hat{x}}_{k+1|k+1}]y_{k+1}^{T}\\ =E_{x}E_{\gamma }\{A_{k}[x_{k}-{\hat{x}}_{k|k}]-K_{k+1}J[x_{k}-{\hat{x}}_{k|k}]+K_{k+1}{\mathrm{Jx}}_{k}B_{k}w_{k}\\ -K_{k+1}[[\gamma_{k+1}C_{k+1}^{j_{k+1}}A_{k}+(1-\gamma_{k+1})\gamma_{k}C_{k}^{j_{k}}+(1-\gamma_{k+1})(1-\gamma_{k})C_{k-1}^{j_{k-1}}A_{k-1}^{-1}]x_{k}\\ +[\gamma_{k+1}v_{k+1}^{j_{k+1}}+(1-\gamma_{k+1})\gamma_{k}v_{k}^{j_{k}}+(1-\gamma_{k+1})(1-\gamma_{k})v_{k-1}^{j_{k-1}}]+[\gamma_{k+1}C_{k+1}^{j_{k+1}}B_{k}w_{k}-(1-\gamma_{k+1})(1-\gamma_{k})C_{k-1}^{j_{k-1}}A_{k-1}^{-1}B_{k-1}w_{k-1}]\}\\ \times \{[\gamma_{k+1}C_{k+1}^{j_{k+1}}A_{k}+(1-\gamma_{k+1})\gamma_{k}C_{k}^{j_{k}}+(1-\gamma_{k+1})(1-\gamma_{k})C_{k-1}^{j_{k-1}}A_{k-1}^{-1}]x_{k}\\ +{\left[\gamma_{k+1}v_{k+1}^{j_{k+1}}+(1-\gamma_{k+1})\gamma_{k}v_{k}^{j_{k}}(1-\gamma_{k+1})(1-\gamma_{k})v_{k-1}^{j_{k-1}}]+[\gamma_{k+1}C_{k+1}^{j_{k+1}}B_{k}w_{k}-(1-\gamma_{k+1})(1-\gamma_{k})C_{k-1}^{j_{k-1}}A_{k-1}^{-1}B_{k-1}w_{k-1}]\right\}}^{T}\\ =A_{k}P_{k|k}J^{T}-K_{k+1}{\mathrm{JP}}_{k|k}J^{T}+K_{k+1}{\mathrm{JS}}_{k}J^{T}-K_{k+1}[p_{k+1}C_{k+1}^{j_{k+1}}A_{k}S_{k}A_{k}^{T}{\left(C_{k+1}^{j_{k+1}}\right)}^{T}\\ +(1-p_{k+1})p_{k}C_{k}^{j_{k}}S_{k}{\left(C_{k}^{j_{k}}\right)}^{T}+(1-p_{k+1})(1-p_{k})C_{k-1}^{j_{k-1}}A_{k-1}^{-1}S_{k}A_{k-1}^{-T}{\left(C_{k-1}^{j_{k-1}}\right)}^{T}]\\ +p_{k+1}B_{k}Q_{k}B_{k}^{T}{\left(C_{k+1}^{j_{k+1}}\right)}^{T}-K_{k+1}[p_{k+1}R_{k+1}^{j_{k+1}}+(1-p_{k+1})p_{k}R_{k}^{j_{k}}+(1-p_{k+1})(1-p_{k})R_{k-1}^{j_{k-1}}\\ +p_{k+1}C_{k+1}^{j_{k+1}}B_{k}Q_{k}B_{k}^{T}{\left(C_{k+1}^{j_{k+1}}\right)}^{T}+(1-p_{k+1})(1-p_{k})C_{k-1}^{j_{k-1}}A_{k-1}^{-1}B_{k-1}Q_{k-1}B_{k-1}^{T}A_{k-1}^{-T}{\left(C_{k-1}^{j_{k-1}}\right)}^{T}]\\ =0 \end{array}$$

From the above equation it may derive (15).

According to (11) in Lemma 1, it follows that:

$$\begin{array}{l} P_{k+1|k+1}\\ =E_{x}E_{\gamma }[x_{k+1}-{\hat{x}}_{k+1|k+1}]x_{k+1}^{T}\\ =E_{x}E_{\gamma }\{A_{x}(x_{k}-{\hat{x}}_{k|k})-K_{k+1}J(x_{k}-{\hat{x}}_{k|k})+K_{k+1}Jx_{k}+B_{k}w_{k}\\ -K_{k+1}[[\gamma_{k+1}C_{k+1}^{j_{k+1}}A_{k}+(1-\gamma_{k+1})\gamma_{k}C_{k}^{j_{k}}+(1-\gamma_{k+1})(1-\gamma_{k})C_{k-1}^{j_{k-1}}A_{k-1}^{-1}]x_{k}\\ \;\;\;\;\;+\gamma_{k+1}v_{k+1}^{j_{k+1}}+(1-\gamma_{k+1})\gamma_{k}v_{k}^{j_{k}}+(1-\gamma_{k+1})(1-\gamma_{k})v_{k-1}^{j_{k-1}}\\ +\gamma_{k+1}C_{k+1}^{j_{k+1}}B_{k}w_{k}-(1-\gamma_{k+1})(1-\gamma_{k})C_{k-1}^{j_{k-1}}A_{k-1}^{-1}B_{k-1}w_{k-1}]\}\times {\left\{A_{k}x_{k}+B_{k}w_{k}\right\}}^{T}\\ =(A_{k}-K_{k+1}J)P_{k|k}A_{k}^{T}+(I_{n}-p_{k+1}K_{k+1}C_{k+1}^{j_{k+1}})B_{k}Q_{k}B_{k}^{T} \end{array}$$

where Assumption 1 is used and *I_n_* is *n* × *n* identity matrix.

From Definition, the next equation is derived:

$$S_{k+1}=E[x_{k+1}x_{k+1}^{T}]=A_{k}S_{k}A_{k}^{T}+B_{k}Q_{k}B_{K}^{T}$$

**Remark 2:**

In special case 1, when packet arrival rate *p*_*k*+1_ = 0 and *p_k_* = 0:

(23a) $$F_{k+1}=A_{k}-K_{k+1}C_{k-1}^{j_{k-1}}A_{k-1}^{-1}$$

(23b) $$K_{k+1}=A_{k}P_{k|k}A_{k-1}^{-T}{\left(C_{k-1}^{j_{k-1}}\right)}^{T}{\left[C_{k-1}A_{k-1}^{-1}P_{k|k}A_{k-1}^{-T}{\left(C_{k-1}^{j_{k-1}}\right)}^{T}+R_{k-1}^{j_{k-1}}+C_{k-1}^{j_{k-1}}A_{k-1}^{-1}B_{k-1}Q_{k}B_{k-1}^{T}A_{k-1}^{-1}{\left(C_{k-1}^{j_{k-1}}\right)}^{T}\right]}^{-1}$$

From (23) we know that the information at the current step and the information at the latest previous step are consecutively lost. In this situation, the filter (13) uses the (*k* − 1)*^th^* step measurement to update the information of the (*k* + 1)*^th^* step.

In special case 2, when packet arrival rate *p*_*k*+1_ = 0 and *p_k_* = 1:

(24a) $$F_{k+1}=A_{k}-K_{k+1}C_{k}^{j_{k}}$$

(24b) $$K_{k+1}=A_{k}P_{k|k}{\left(C_{k}^{j_{k}}\right)}^{T}{\left[C_{k}^{j_{k}}P_{k|k}{\left(C_{k}^{j_{k}}\right)}^{T}+R_{k}^{j_{k}}\right]}^{-1}$$

It means that the information at the current step is lost. In this situation, the filter (13) uses the *k^th^* step measurement to update information of the (*k* + 1)*^th^* step.

In special case 3, when packet arrival rate *p*_*k*+1_ = 1:

(25a) $$F_{k+1}=A_{k}-K_{k+1}C_{k+1}^{j_{k+1}}A_{k}$$

(25b) $$\begin{array}{ll} K_{k+1} & =(A_{k}P_{k|k}A_{k}^{T}+B_{k}Q_{k}B_{k}^{T}){\left(C_{k+1}^{j_{k+1}}\right)}^{T}\\ & \;\;\;\;\times {\left[C_{k+1}^{j_{k+1}}A_{k}P_{k|k}A_{k}^{T}{\left(C_{k+1}^{j_{k+1}}\right)}^{T}+R_{k+1}^{j_{k+1}}+C_{k+1}^{j_{k+1}}B_{k}Q_{k}B_{k}^{T}{\left(C_{k+1}^{j_{k+1}}\right)}^{T}\right]}^{-1} \end{array}$$

In this situation, the LMVF reduces the standard KF. An example for DTSL system is given in the following part.

### 3.2. An example for LMVF

We give a numerical example to verify the validity of Theorem 1. It is assumed $C_{k}^{j_{k}}$ is a constant in the following equation at each time step and:

(26a) $$x_{k+1}=A_{k}x_{k}+B_{k}w_{k}$$

(26b) $$z_{k}^{j_{k}}=C_{k}^{j_{k}}x_{k}+v_{k}^{j_{k}}$$

(26c) $$y_{k}=\gamma_{k}z_{k}^{j_{k}}+(1-\gamma_{k})\gamma_{k-1}z_{k-1}^{j_{k-1}}+(1-\gamma_{k})(1-\gamma_{k-1})z_{k-2}^{j_{k-2}}$$

where $A_{k}=\left[\begin{array}{cc} 0.6 & 0\\ 0.8 & 0.5 \end{array}\right]$, $B_{k}=\left[\begin{array}{c} 0.6\\ 0.5 \end{array}\right]$, $C_{k}^{j_{k}}=\left[\begin{array}{cc} 1 & 1 \end{array}\right]$ and $x_{k}={\left[\begin{array}{cc} x_{k}^{1} & x_{k}^{2} \end{array}\right]}^{T}$.

Define estimation error ${\mathrm{Er}}_{k}^{i}$ at time step *k* and total estimation error *Er* respectively:

(27) $${\mathrm{Er}}_{k}^{1}=\left|x_{k}^{1}-{\hat{x}}_{k|k}^{1}\right|,{\mathrm{Er}}_{k}^{2}=\left|x_{k}^{2}-{\hat{x}}_{k|k}^{2}\right|$$

(28) $${\mathrm{Er}}^{1}=\sum_{k=1}^{N}{\mathrm{Er}}_{k}^{1},{\mathrm{Er}}^{2}=\sum_{k=1}^{N}{\mathrm{Er}}_{k}^{2}$$

where ( ${\left({\hat{x}}_{k|k}^{1},{\hat{x}}_{k|k}^{2}\right)}^{T}$ is the estimate of *x_k_*; *N* denotes total time step.

**Figure 2.**
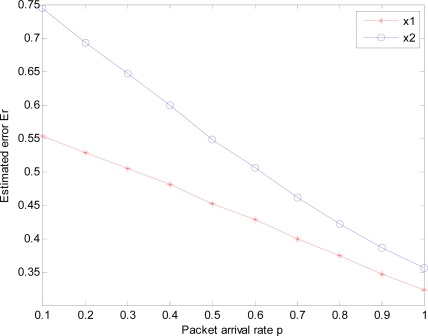
Estimation error of LMVFs under different packet arrival rate 0.1 ≤ *p* ≤ 1 based on 100 Monte Carlo simulations.

In simulation, *x̂*_0|0_ = [0 0]^T^, *P*_0|0_ = *diag*([0.1 0.1]), *S*_0_ = *P*_0|0_, *n* = 1,000, $R_{k}^{j_{k}}=1$ and $Q_{k}=\left[\begin{array}{cc} 0.36 & 0.30\\ 0.30 & 0.25 \end{array}\right]$. ${\left({\hat{x}}_{k|k}^{1},{\hat{x}}_{k|k}^{2}\right)}^{T}$ at each time step be calculated making use of Theorem 1. Estimation error *Er_k_* at each time step and total estimation error *Er* are computed by (27) and (28) respectively. 100 Monte Carlo simulations are tested and corresponding simulation results are shown in Figure 2, where the red line with symbol ‘*’ denotes total error *Er*^1^ and the blue line with symbol ‘o’ denotes total error *Er*^2^. From Figure 2 we see the estimation error decreases as packet arrival rate *p* increases from 0.1 to 1. When *p* = 0.1, *Er*^1^ = 0.5527 and *Er*^2^ = 0.7445; When *p*=1, *Er*^1^ = 0.3231 and *Er*^2^ = 0.3557.

The experience results show estimation error is very small. The simulation verifies feasibility and effectiveness of the proposed LMVF.

## 4. Extended Minimum Variance Filter with Multiple Packet Losses

In this section we will show our augmented results on extended minimum variance filters with multiple packet losses. In time-varying DTSN systems, an extended minimum variance filter is to be derived and it is extended to nonlinear case by Taylor series approximation in the following section.

### 4.1. Design of Extended Minimum Variance Filter with Multiple Packet Losses

In mobile target tracking of WSNs, the state models of the plant and measurement models are usually nonlinear, so we require making linearization for models.

Rewriting (1), let it become DTSN system:

(29a) $$x_{k+1}=\varphi (x_{k})+B_{k}w_{k}$$

(29b) $$z_{k}^{j_{k}}=h^{j_{k}}(x_{k})+v_{k}^{j_{k}}$$

(29c) $$y_{k}=\gamma_{k}z_{k}^{j_{k}}+(1-\gamma_{k})\gamma_{k-1}z_{k-1}^{j_{k-1}}+(1-\gamma_{k})(1-\gamma_{k-1})z_{k-2}^{j_{k-2}}$$

where *φ*(*x_k_*) and *h*(*x_k_*) are nonlinear functions with respective to the state *x_k_* and the time step *k*. The other conditions are similar to (1a) and (1b) in Section 2.

To obtain the estimated state *x̂*_*k*+1|*k*+1_, the nonlinear function in (29a) is expanded in Taylor series around the latest estimate *x̂*_*k*|*k*_ with the first order to yield an extended minimum variance filter. Taylor series expansion of (29a) to the first order is approximately:

(30) $$x_{k+1}=\varphi ({\hat{x}}_{k|k})+\Phi_{k}(x_{k}-{\hat{x}}_{k|k})+B_{k}w_{k}$$

where $\Phi_{k}={\frac{\partial \phi \left(x_{k}\right)}{\partial x_{k}}|}_{x_{k}={\hat{x}}_{k|k}}$ is the Jacobian matrix of the function *φ*(*x_k_*).

Similarly, we make linearization of the nonlinear measurement function (29c) by Taylor series:

(31) $$\begin{array}{l} y_{k}=\gamma_{k}h^{j_{k}}({\hat{x}}_{k|k-1})+\gamma_{k}H_{k}^{j_{k}}(x_{k}-{\hat{x}}_{k|k-1})+\gamma_{k}v_{k}^{j_{k}}\\ +(1-\gamma_{k})\gamma_{k-1}h^{j_{k}}({\hat{x}}_{k-1|k-2})+(1-\gamma_{k})\gamma_{k-1}H_{k-1}^{j_{k-1}}(x_{k-1}-{\hat{x}}_{k-1|k-2})+(1-\gamma_{k})\gamma_{k-1}v_{k-1}^{j_{k-1}}\\ +(1-\gamma_{k})(1-\gamma_{k-1})h^{j_{k-2}}({\hat{x}}_{k-2|k-3})+(1-\gamma_{k})(1-\gamma_{k-1})H_{k-2}^{j_{k-2}}(x_{k-2}-{\hat{x}}_{k-2|k-3})+(1-\gamma_{k})(1-\gamma_{k-1})v_{k-2}^{j_{k-2}} \end{array}$$

where $H_{k}^{j_{k}}={\frac{{\partial h}^{j_{k}}\left(x_{k}\right)}{{\partial x}_{k}}|}_{x_{k}={\hat{x}}_{k|k-1}}$ is the Jacobian matrix of the function *h*^*j_k_*^ (•). Based on (30) and (31), Theorem 2 is shown as follows:

**Theorem 2**: For nonlinear systems (30) and (31) satisfying Assumption 1 and 2, extended minimum variance filter (EMVF) is shown as follows:

(32) $$\begin{array}{ll} {\hat{x}}_{k+1|k+1}= & {\hat{x}}_{k+1|k}+K_{k+1}[y_{k+1}-p_{k+1}h^{j_{k+1}}({\hat{x}}_{k+1|k})\\ & \;\;\;-(1-p_{k+1})p_{k}h^{j_{k}}({\hat{x}}_{k|k-1})-(1-p_{k+1})(1-p_{k})h^{j_{k-1}}({\hat{x}}_{k-1|k-2})] \end{array}$$

where:

(33) $${\hat{x}}_{k+1|k}=\varphi ({\hat{x}}_{k|k},k)$$

(34) $$\begin{array}{l} K_{k+1}=[\Phi_{k}P_{k|k}J^{T}+p_{k+1}B_{k}Q_{k}B_{k}^{T}{\left(H_{k+1}^{j_{k+1}}\right)}^{T}]\times \{{\mathrm{JP}}_{k|k}J^{T}-{\mathrm{JS}}_{k}J^{T}+p_{k+1}H_{k+1}^{j_{k+1}}\Phi_{k}S_{k}\Phi_{k}^{T}{\left(H_{k+1}^{j_{k+1}}\right)}^{T}\\ +p_{k}(1-p_{k+1})H_{k}^{j_{k}}S_{k}{\left(H_{k}^{j_{k}}\right)}^{T}+(1-p_{k})(1-p_{k+1})H_{k-1}^{j_{k-1}}\Phi_{k-1}^{-1}S_{k}\Phi_{k-1}^{-T}{\left(H_{k-1}^{j_{k-1}}\right)}^{T}\\ +p_{k+1}R_{k+1}^{j_{k+1}}+p_{k}(1-p_{k+1})R_{k}^{j_{k}}+(1-p_{k})(1-p_{k+1})R_{k-1}^{j_{k-1}}\\ {+(1-p_{k})(1-p_{k+1})H_{k-1}^{j_{k-1}}\Phi_{k-1}^{-1}B_{k-1}Q_{k-1}B_{k-1}^{T}\Phi_{k-1}^{-T}{\left(H_{k-1}^{j_{k-1}}\right)}^{T}+p_{k+1}H_{k+1}^{j_{k+1}}B_{k}Q_{k}B_{k}^{T}{\left(H_{k+1}^{j_{k+1}}\right)}^{T}\}}^{-1} \end{array}$$

(35) $$P_{k+1|k+1}=(\Phi_{k}-K_{k+1}J)P_{k|k}\Phi_{k}^{T}+(I_{n}-p_{k+1}K_{k+1}H_{k+1}^{j_{k+1}})B_{k}Q_{k}B_{k}^{T}$$

(36) $$J=p_{k+1}H_{k+1}^{j_{k+1}}\Phi_{k}+p_{k}(1-p_{k+1})H_{k}^{j_{k}}+(1-p_{k})(1-p_{k+1})H_{k-1}^{j_{k-1}}\Phi_{k-1}^{-1}$$

(37) $$S_{k+1}=\Phi_{k}S_{k}\Phi_{k}^{T}+B_{k}Q_{k}B_{k}^{T}$$

with:

(38) $${\hat{x}}_{0|-1}=m_{0},P_{0|0}=P_{0}\;\text{and}\;S_{0}=P_{0}$$

The proof of Theorem 2 is similar to Theorem 1, so it is omitted here. Theorem 2 can be applied to nonlinear cases of the measurement model and state model. An application example for EMVF is given in the following subsection.

### 4.2. Application of EMVF in WSNs

In the above subsection a kind of EMVF is designed. In the following we apply the EMVF to track mobile target in WSNs. Usually multiple sensors are scheduled cooperatively to complete a common task. Firstly we give a practice state model and measurement model, and then provide a sensor scheduling strategy. At last, simulation results illustrate our EMVF is feasible and effective in WSNs.

#### 4.2.1 Nonlinear state model

We use the nonlinear state model of reference [20]. The idea is to augment the state vector with a turning rate parameter *ω*, which is to be estimated along with the other system parameters, which in this example are the position $\left(x_{k}^{t},y_{k}^{t}\right)$ and the velocity $\left({\dot{x}}_{k}^{t},{\dot{y}}_{k}^{t}\right)$ of the target. Thus, the state vector can be expressed as:

(39) $$x_{k}={\left[\begin{array}{ccccc} x_{k}^{t} & y_{k}^{t} & {\dot{x}}_{k}^{t} & {\dot{y}}_{k}^{t} & \omega_{k}^{t} \end{array}\right]}^{T}$$

and the dynamic model for the coordinated turns is:

(40) $$x_{k+1}={\overset{⌢}{A}}_{k}x_{k}+B_{k}w_{k}$$

where:

$$\begin{array}{c} {\overset{⌢}{A}}_{k}=\left[\begin{array}{ccccc} 1 & 0 & \frac{\text{sin}(\Delta t_{k}\omega_{k}^{t})}{\omega_{k}^{t}} & \frac{\text{cos}(\Delta t_{k}\omega_{k}^{t})-1}{\omega_{k}^{t}} & 0\\ 0 & 1 & \frac{\text{cos}(\Delta t_{k}\omega_{k}^{t})-1}{\omega_{k}^{t}} & \frac{\text{sin}(\Delta t_{k}\omega_{k}^{t})}{\omega_{k}^{t}} & 0\\ 0 & 0 & \text{cos}(\Delta t_{k}\omega_{k}^{t}) & -\text{sin}(\Delta t_{k}\omega_{k}^{t}) & 0\\ 0 & 0 & \text{sin}(\Delta t_{k}\omega_{k}^{t}) & \text{cos}(\Delta t_{k}\omega_{k}^{t}) & 0\\ 0 & 0 & 0 & 0 & 1 \end{array}\right]\\ B_{k}={\left[\begin{array}{ccccc} 0 & 0 & 0 & 0 & 1 \end{array}\right]}^{T} \end{array}$$

and *w_k_* is white Gaussian process noise with zero mean and variance *Q_k_*. The Jacobian matrix of *Ȃ_k_* is computed as:

$$\Phi_{k}=\left[\begin{array}{ccccc} 1 & 0 & \frac{\text{sin}(\Delta t_{k}\omega_{k}^{t})}{\omega_{k}^{t}} & \frac{\text{cos}(\Delta t_{k}\omega_{k}^{t})-1}{\omega_{k}^{t}} & \frac{{\partial x}_{k+1}^{t}}{{\partial \omega }_{k}^{t}}\\ 0 & 1 & \frac{\text{cos}(\Delta t_{k}\omega_{k}^{t})-1}{\omega_{k}^{t}} & \frac{\text{sin}(\Delta t_{k}\omega_{k}^{t})}{\omega_{k}^{t}} & \frac{{\partial y}_{k+1}^{t}}{{\partial \omega }_{k}^{t}}\\ 0 & 0 & \text{cos}(\Delta t_{k}\omega_{k}^{t}) & -\text{sin}(\Delta t_{k}\omega_{k}^{t}) & \frac{{\partial \dot{x}}_{k+1}^{t}}{{\partial \omega }_{k}^{t}}\\ 0 & 0 & \text{sin}(\Delta t_{k}\omega_{k}^{t}) & \text{cos}(\Delta t_{k}\omega_{k}^{t}) & \frac{{\partial \dot{y}}_{k+1}^{t}}{{\partial \omega }_{k}^{t}}\\ 0 & 0 & 0 & 0 & 1 \end{array}\right]$$

where:

$$\begin{array}{c} \frac{{\partial x}_{k+1}^{t}}{{\partial \omega }_{t}^{k}}=\frac{\Delta t_{k}\omega_{k}^{t}\text{cos}(\Delta t_{k}\omega_{k}^{t})+\text{sin}(\Delta t_{k}\omega_{k}^{t})-1}{{\left(\omega_{k}^{t}\right)}^{2}}{\dot{x}}_{k}-\frac{\Delta t_{k}\omega_{k}^{t}\text{sin}(\Delta t_{k}\omega_{k}^{t})+\text{cos}(\Delta t_{k}\omega_{k}^{t})}{{\left(\omega_{k}^{t}\right)}^{2}}{\dot{y}}_{k}\\ \frac{{\partial y}_{k+1}^{t}}{{\partial \omega }_{k}^{t}}=\frac{\Delta t_{k}\omega_{k}^{t}\text{sin}(k\omega_{k}^{t})+\text{cos}(\Delta t_{k}\omega_{k}^{t})-1}{{\left(\omega_{k}^{t}\right)}^{2}}{\dot{x}}_{k}-\frac{k\omega_{k}^{t}\text{cos}(\Delta t_{k}\omega_{k}^{t})+\text{sin}(\Delta t_{k}\omega_{k}^{t})}{{\left(\omega_{k}^{t}\right)}^{2}}{\dot{y}}_{k}\\ \frac{{\partial \dot{x}}_{k+1}^{t}}{{\partial \omega }_{k}^{t}}=\Delta t_{k}\text{sin}(\Delta t_{k}\omega_{k}^{t}){\dot{x}}_{k}-\Delta t_{k}\text{cos}(\Delta t_{k}\omega_{k}^{t}){\dot{y}}_{k}\\ \frac{{\partial \dot{y}}_{k+1}^{t}}{{\partial \omega }_{k}^{t}}=\Delta t_{k}\text{cos}(\Delta t_{k}\omega_{k}^{t}){\dot{x}}_{k}-\Delta t_{k}\text{sin}(\Delta t_{k}\omega_{k}^{t}){\dot{y}}_{k} \end{array}$$

#### 4.2.2. Nonlinear measurement model

Sonic sensors are used to measurement target and the measurement model for sensor *j_k_* at time step *k* is defined as follows:

(41) $$\begin{array}{ll} z_{k}^{j_{k}} & =h^{j_{k}}(x_{k})+v_{k}^{j_{k}}\\ & =\sqrt{{\left(x_{k}^{t}-x^{j_{k}}\right)}^{2}+{\left(y_{k}^{t}-y^{j_{k}}\right)}^{2}}+v_{k}^{j_{k}} \end{array}$$

where *j_k_* = 1,2,⋯, *N*, and *N* is the total number of sensor nodes; $\left(x_{k}^{t},y_{k}^{t}\right)$ is the dynamic location of the mobile target and (*x^j_k_^, y^j_k_^*) is the known position of sensor *j_k_* ; $v_{k}^{j_{k}}$ is the zero-mean Gaussian measurement noise with variance $R_{k}^{j_{k}}$ for sensor *j_k_* at the time step *k*; *γ_k_* is a random variable with *Eγ_k_* = *p_k_*.

Since measurement model is nonlinear function, it has to be linearized and Jacobian matrix $H_{k}^{j_{k}}$ of $h_{k}^{j_{k}}$ is computed as follows:

$$H_{k}^{j_{k}}=\left[\begin{array}{cccc} \frac{x_{k}^{t}-x^{j_{k}}}{\sqrt{{\left(x_{k}^{t}-x^{j_{k}}\right)}^{2}+{\left(y_{k}^{t}-y^{j_{k}}\right)}^{2}}} & \frac{y_{k}^{t}-y^{j_{k}}}{\sqrt{{\left(x_{k}^{t}-x^{j_{k}}\right)}^{2}+{\left(y_{k}^{t}-y^{j_{k}}\right)}^{2}}} & 0 & 0 \end{array}\right]$$

#### 4.2.3. Distributed wireless sensor scheduling

In target tracking application of distributed WSNs, there are a number of wireless sensor nodes deployed randomly in a monitored field. One node or several nodes are scheduled as task nodes in target tracking application at each time step *k*.

We improve dynamic-group scheduling strategy (DGSS) [19]. The improved DGSS (IDGSS) is regarded as the scheduler to select next task sensor node during mobile target tracking, where predicted accuracy, predicted energy and real time are considered. We define cost function:

(42) $$J(j_{k},\Delta t_{k})=w\emptyset_{k}^{j_{k}}+(1-w)\frac{E(j_{k},j_{k+1})}{\Delta t_{k}}$$

where $\emptyset_{k}^{j_{k}}$ is the predicted accuracy, *E*(*j_k_*, *j_k_*_+1_) is the predicted energy, Δ*t_k_* is sample interval and *w* ∈ (0,1) is weight parameter used to balance the tracking accuracy and energy consumption.

The task sensor node is scheduled in the following two situations:

After prediction, if none of the sensors can achieve the satisfactory tracking accuracy using any sampling interval between *T*_max_ and *T*_min_, in this case, Δ*t_k_* is set to the minimal sampling interval *T*_min_, and the sensor is selected by:

(43) $$j_{k}={\text{arg}}_{j_{k}\in G}\text{min}\{J(j_{k},T_{\text{min}})\}$$

where *G* is the set of candidate sensors in the group which can be selected by sensor *j_k_*. The purpose of this mode is to drive the tracking accuracy to be satisfactory as soon as possible with consideration of the energy consumption.

After prediction, if at least one sensor can achieve the satisfactory tracking accuracy. In this case, the optimal (*j_k_*, Δ*t_k_*) are selected by:

(44) $$J(j_{k},\Delta t_{k})={\text{arg}}_{j_{k}\in G*,\phi (j_{k},j_{k+1})\leq \phi_{0}}\text{min}\{\frac{E(j_{k},j_{k+1})}{\Delta t_{k}}\}$$

where G^*^ is the set of sensors that can achieve the satisfactory tracking accuracy, and ∅_0_ is the threshold of tracking accuracy. Equation (44) above utilizes the objective function (42) with *w*=0. The basic idea of this mode is that when the predicted tracking accuracy is satisfactory, the sensors and the sampling interval are selected according to the energy efficiency.

The sample interval significantly affects tracking accuracy and energy efficiency of the whole network. For example, with a short sampling interval, the target can be tracked more accurately but lead to much energy consumption. While long sampling interval maybe load tracking accuracy to decrease or the target to be lost. In this paper we suppose the sampling interval is selected from a predefined *N* values ${\left\{T_{t}\right\}}_{1}^{N}$, where *T_1_*=*T_min_*, *T_2_*=*T_max_*, and *T*_*t*1_ < *T*_*t*2_ if *t*_1_ < *t*_2_.

The operations of each task sensor node mainly include the following steps:

1. Measuring the distance $z_{k}^{j_{k}}$ between the motion target and the current sensor node *j_k_* at the current time *k*, where (41) is used;
2. Performing extended minimum variance filtering algorithm with packet losses by Theorem 2 in WSNs, and calculating the prediction accuracy according to $\emptyset_{k}^{j_{k}}=\left[\begin{array}{ccccc} 1 & 1 & 0 & 0 & 0 \end{array}\right]P_{k|k}{\left[\begin{array}{ccccc} 1 & 1 & 0 & 0 & 0 \end{array}\right]}^{T}$;
3. Transmitting the predicted accuracy $\emptyset_{k}^{j_{k}}$ and sample interval Δ*t_k_* according to (43) and (44) at time step *k* to the scheduler.

We suppose that all sensor nodes are usually in the sleeping mode and are awaked to perform sensing tasks by using an ultralow power channel when they are scheduled to perform the sensing tasks.

**Figure 3.**
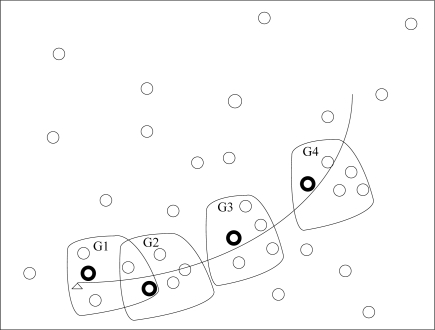
The framework of IDGSS.

The scheduler of IDGSS is shown as follows:

- First, the nearest node to the target (such as bold small circle in the group G1 in Figure 3) is awaked from sleep state and regarded as the task node at current time step *k* in the neighbor region;
- The nearest sensor node is considered as the center node in a local neighbor area, total *M* nearest nodes to the target are awaked to form a group. When the target lies in the group, we consider both estimation accuracy and energy. If estimation accuracy is satisfactory, the sensor node, whose consumption energy is least, is selected to track the target by (44) at the next time step *k*+1, and changed sample interval Δ*t_k_* is used in order to save energy; If estimation accuracy is not satisfactory, (43) is used to select next task node *j_k_* and *T*_min_ is used as sample interval;
- When the target moves out of the group, the sensor nodes in the group return sleep state, and a new task node is awaked from the sleep state in a new local neighbor region. Similarly, a new dynamic group is formed again, as seen from Figure 3, such as G2, G3, G4,…

**Remark 3**: we use dynamic grouping idea and firstly awake M sensor nodes in order to increase real-time performance of target tracking, and adopt the changed sampling intervals during target tracking in order to save energy consumption under the satisfactory estimation accuracy.

#### 4.2.4. Simulation results

Nonlinear state model and measurement model in Section 4.2.1 and 4.2.2 are adopted to track the mobile target in WSNs.

Define two measurements received as follows respectively.

*Model 1*:

(45a) $$y_{k}^{1}=\gamma_{k}z_{k}^{j_{k}}+(1-\gamma_{k})\gamma_{k-1}z_{k-1}^{j_{k-1}}$$

*Model 2*:

(45b) $$y_{k}^{2}=\gamma_{k}z_{k}^{j_{k}}+(1-\gamma_{k})\gamma_{k-1}z_{k-1}^{j_{k-1}}+(1-\gamma_{k})(1-\gamma_{k-1})z_{k-2}^{j_{k-2}}$$

where $y_{k}^{1}$ denotes that the filter uses the previous measurement $z_{k-1}^{j_{k-1}}$ to update if the current measurement $z_{k}^{j_{k}}$ is lost, and the filter uses only zero to update if both the current measurement and the previous measurement are lost at time step *k*; Different from $y_{k}^{1}$, $y_{k}^{2}$ denotes that the filter can use $z_{k-2}^{j_{k-2}}$ to update when both the current measurement $z_{k}^{j_{k}}$ and the previous measurement $z_{k-1}^{j_{k-1}}$ are lost at time step *k*.

**Figure 4.**
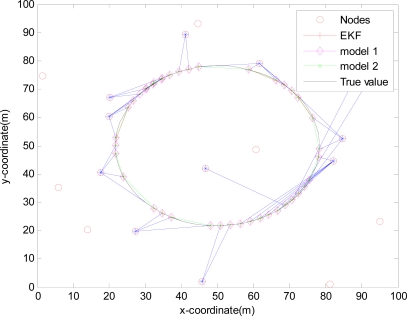
Real trajectory and estimation with IDGSS under packet arrival rate *p* = 1.

**Figure 5.**
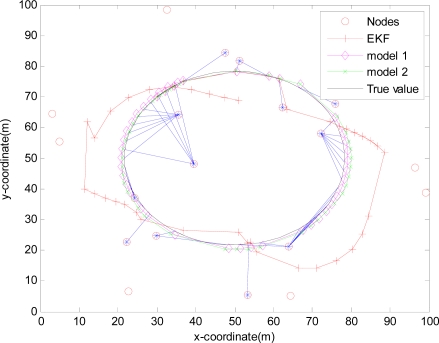
Real trajectory and estimation with IDGSS under packet arrival rate *p* = 0.98.

**Figure 6.**
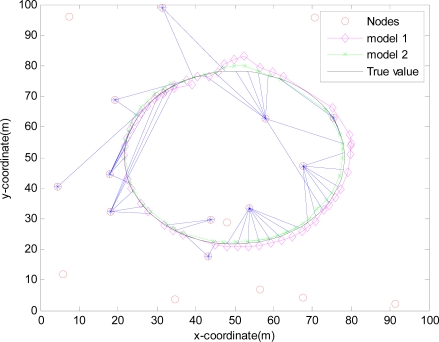
Real trajectory and estimation with IDGSS under packet arrival rate *p* = 0.95.

**Figure 7.**
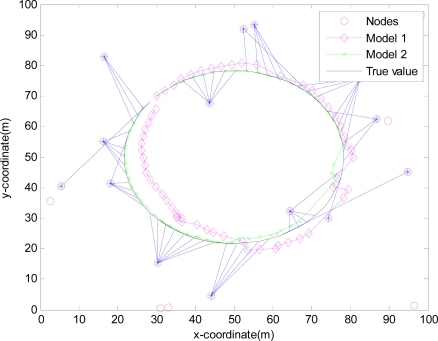
Real trajectory and estimation with IDGSS under packet arrival rate *p* = 0.90.

The monitored field is 100 m×100 m and covered by 20 sensors randomly deployed in Figures 4, 5, 6 and 7, where the IDGSS is adopted in the target tracking, and the red circles represent random sensor nodes deployed in the monitored WSNs area, and the red circles with symbol ‘*’ represent sensor nodes scheduled in the process of the target tracking, and the associated tasking sensor of each estimated target position is indicated by a blue line between them.

**Figure 8.**
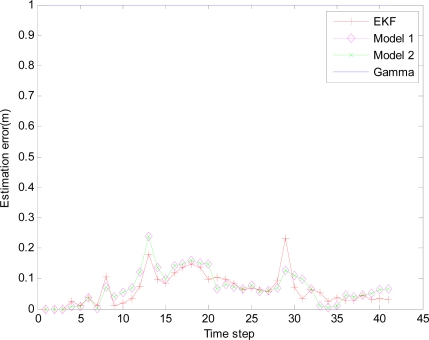
Estimation error with IDGSS under packet arrival rate *p* = 1.

**Figure 9.**
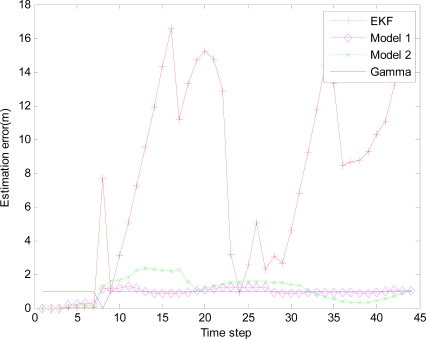
Different Filters with IDGSS under packet arrival rate *p* = 0.98.

**Figure 10.**
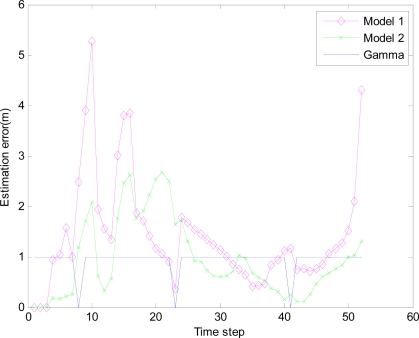
Estimation error of different filters with IDGSS under packet arrival rate *p* = 0.95.

**Figure 11.**
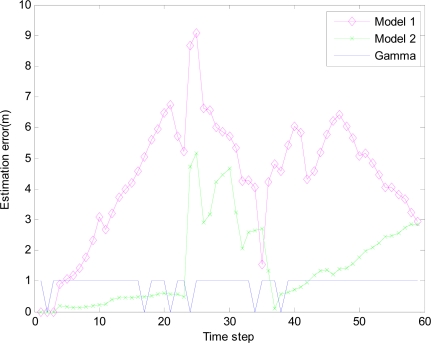
Estimation error of different filters with IDGSS under packet arrival rate *p* = 0.90.

In the monitored field the target moves along the circle trajectory, whose start position is (30, 70). For Theorem 2, initial state vector *x̂*_0|−1_ =[30 70 20 20 −1]^T^, initial covariance matrix *P*_0|0_ =2×*diag*([0.1 0.1 0.1 0.1 0.1]) and initial variance *S*_0_ = *P*_0|0_ in (38). Total time step *N* = 67, number of sensor in a group *M* = 5, *q* = 0.2 *R_k_* = 0.001 and *Q_k_* = *B_k_* × *q* × *B_k_^T^*, where *B_k_* = [0 0 0 0 0.1]*^T^*. Minimum (maximum) time sampling interval *T_min_* = 0.1 (*T_max_* = 0.5). Weight parameter *w* = 0.7. Threshold of accuracy ∅_0_ = 8. The estimation errors of the EMVF under different packet arrival rate are shown in Figures 8, 9, 10 and 11, where estimation error is defined as the distance between the estimated target positions and the true position of the target, and *γ_k_* (Gamma) is a random variable taking value 0 or 1.

For simplicity, in this simulation packet arrival rate *p_k_* = *p*. In Figure 4 when packet arrival rate *p* = 1, standard EKF, *Model 1* and *Model 2* have the approximately same tracking trajectory. Estimation error is approximate in Figure 8. Because packet arrival rate *p* = 1, we can verify that corresponding EMVF of Model *1* and *Model 2* reduce standard EKF [20]. When packet loss rate *p* = 0.98, standard EKF starts to diverge once packet loss happens, while *Model 1* and *Model 2* still can track the mobile target in Figure 5. When the time step *k* = 8, packet loss happens, and then EKF has much higher estimation error than *Model 1* and *Model 2* in Figure 9. When packet arrival rate *p* is 0.95 in Figure 6, compared with *Model 1*, *Model 2* has superior tracking trajectory. Moreover, *Model 2* shows smaller estimation error than *Model 1* in Figure 10. *Model 2* has better performance than *Model 1*, because *Model 1* can’t deal with consecutive two measurements dropped, while *Model 2* still can work well. It is similar in Figure 7 and Figure 11. Moreover, From Figure 8 to Figure 11, we know that estimation error becomes more and more small when packet arrival rate *p* increases.

**Figure 12.**
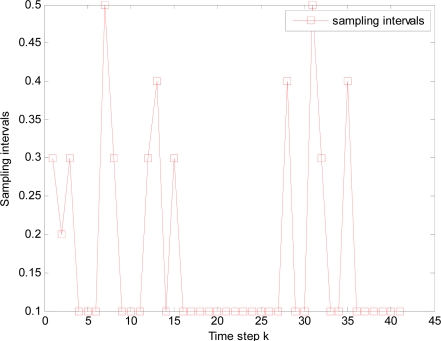
Changed sampling interval under packet arrival rate *p* = 1.

**Figure 13.**
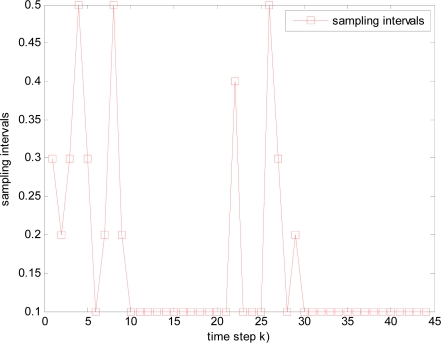
Changed sampling interval under packet arrival rate *p* = 0.98.

**Figure 14.**
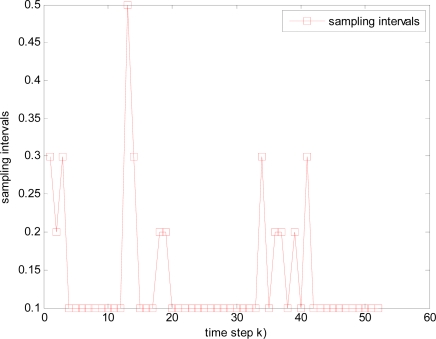
Changed sampling interval under packet arrival rate *p* = 0.95.

It is illustrated that sample interval becomes small and total time steps increase when packet arrival rate *p* decreases, such as the total 42, 44, 52 and 58 time steps in Figure 12, 13, 14, and 15 respectively, where it is also resulted in that consumed energy increases when packet arrival rate *p* decreases in WSNs.

The simulation results verify that derived EMVF is feasible and available in WSNs. Compared with EKF, EMVF has superior performance in tracking mobile target.

In order to illustrate further the effect of consumption energy and estimation accuracy to sampling intervals, we assume that packet arrival rate *p*=1 and estimation accuracy threshold ∅_0_ = 0.5.

Consumed energy (*Ce*) and estimation accuracy (*Ec*) are defined as follows respectively:

$$\begin{array}{c} \mathrm{Ce}=\frac{1}{\mathrm{mn}}\sum_{i=1}^{m}\sum_{k=0}^{n}E(j_{k},j_{k+1})\\ \mathrm{Ec}=\frac{1}{m}\sum_{t=1}^{m}\left(\frac{1}{n}\sum_{k=1}^{n}\emptyset_{k}^{j_{k}}\right) \end{array}$$

Table 1 demonstrates that consumption energy is quite small and estimation accuracy is not also high when IDGSS is adopted. The experience shows that IDGSS make tradeoffs between consumed energy and estimation accuracy during target tracking in WSNs.

## 5. Conclusions

For DTSL systems and DTSN systems, a linear optimal filter and an extended minimum variance filter with packet losses are designed in this paper, respectively. Especially, the proposed EMVF is applied to WSNs for target tracking. A first application example is given and the corresponding simulation result verifies the effectiveness and advantages of the proposed LMVF. The second application example illustrates that the EMVF with multiple packet losses is feasible and available for target tracking in WSNs. In the future, we will further study filters with both multiple time delays and finite consecutive packet losses and in WSNs.

## Figures and Tables

**Figure 15.**
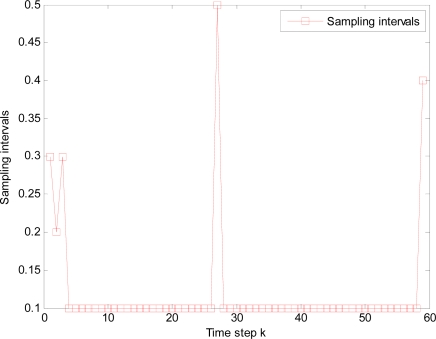
Changed sampling interval under packet arrival rate *p* = 0.90.

**Table 1.** Consumed energy and estimation accuracy under different sampling intervals with IDGSS based on 100 Monte Carlo simulations.

Sampling interval (s)	0.1	0.2	0.3	0.4	0.5	IDGSS
Consumed energy (mJ)	14.8564	9.1176	7.0780	6.1009	5.6857	6.0649
Estimation accuracy(m)	0.3161	0.4407	0.4973	0.6450	0.7114	0.6729
